# Characteristics of the medical malpractice cases against orthopedists in China between 2016 and 2017

**DOI:** 10.1371/journal.pone.0248052

**Published:** 2021-05-12

**Authors:** Hongzhi Lv, Dongzheng Li, Chao Li, Peizhi Yuwen, Zhiyong Hou, Wei Chen, Yingze Zhang

**Affiliations:** 1 Department of Journal Center, The Third Hospital of Hebei Medical University, Shijiazhuang, China; 2 Office of Orthopedic Clinical Medicine Research Center, The Third Hospital of Hebei Medical University, Shijiazhuang, China; 3 Department of Orthopedic Surgery, The Third Hospital of Hebei Medical University, Shijiazhuang, China; University of California San Francisco, UNITED STATES

## Abstract

**Background:**

This study aimed to identify the most frequent reasons for orthopedic medical malpractice, gain insight into the related patient demographics and clinical characteristics, and identify the independent factors associated with it.

**Methods:**

We collected and analyzed the demographic and injury characteristics, hospital levels and treatments, medical errors, and orthopedist’s degree of responsibility for the patients who were subject to orthopedic medical malpractice at our institution. Univariate and multivariate analyses were performed to identify the factors associated with the orthopedist’s degree of responsibility in the medical malpractice cases.

**Results:**

We included 1922 cases of medical malpractice in the final analysis. There were 1195 and 727 men and women, respectively (62.2% and 37.8%, respectively). Of the total patients, 1810, 1038, 1558, 1441, and 414 patients (94.2%, 54.0%, 81.1%, 75.0%, and 21.5%, respectively) were inpatients, had closed injuries, underwent surgery, were trauma cases, and had preoperative comorbidities, respectively. Most medical malpractice cases were in patients with fractures and spinal degenerative disease (1229 and 253 cases; 63.9% and 13.2%, respectively), and occurred in city-level hospitals (1006 cases, 52.3%), which were located in the eastern part of china (1001, 52.1%), including Jiangsu and Zhejiang (279 and 233 cases, 14.52% and 52.1%, respectively). Between 2016 and 2017, the orthopedist’s degree of responsibility in medical malpractice claims were deemed as full, primary, equal, secondary, and minor in 135, 654, 77, 716, and 340 orthopedists (7.0%, 34.0%, 4.0%, 37.3%, and 17.7%). Most medical errors made by orthopedists in cases of medical malpractice were related to failure to supervise or monitor cases, improper performance of procedures, and failure to instruct or communicate with the patient (736, 716, and 423 cases; 38.3%, 37.3%, and 22.0%, respectively). The multivariate analysis found that patients with preoperative comorbidities, who sustained humerus injuries, who were aged ≥65 years, who were treated by doctors who failed to supervise or monitor them, and who were treated at the provincial and city level hospitals were more likely to claim that the orthopedist bore a serious degree of responsibility in the medical malpractice case.

**Conclusions:**

Our results provide detailed information on the plaintiff demographics, clinical characteristics, and factors associated with medical malpractice. Medical malpractice is related to poor treatment outcomes. The first preventative measure that is required is a comprehensive improvement in the medical staff quality, mainly through medical ethics cultivation, and professional ability and technique training. Additionally, failure to supervise or monitor cases was the leading cause of medical malpractice and one of the factors that led to orthopedists bearing an equal and higher responsibility for medical malpractice. Orthopedists should improve patient supervision, especially when treating older patients and those with preoperative comorbidities and humerus injuries.

## Introduction

As the incidence of fractures and degenerative musculoskeletal disease has increased, so has the incidence of orthopedic surgeries [1, 2]. Due to the increasing demand for functional recovery after surgery, rising awareness of patients’ rights, and easy access to patients’ medical information, the number of medical malpractice claims against orthopedists is rising rapidly. Orthopedic surgery has been a topic in medical malpractice for more than 130 years [3]. Medical negligence affects clinicians in all specialties, and orthopedists are the most likely to have lawsuits raised against them. It is estimated that an orthopedic surgeon is exposed to an average of 17 litigation proceedings over their career [4]. Due to the nature of the work, which involves being responsible for patients’ health, medical malpractice may be considered inevitable. Therefore, it is imperative that the quality of orthopedic services be improved through methods such as medical ethics cultivation, professional ability training, and technical improvement. Through a systematic analysis, we can gain a better understanding of orthopedic medical malpractice and adopt preventative strategies. However, detailed information is not widely available to serve as a reminder to orthopedists in China. Therefore, an up-to-date survey on the characteristics of medical malpractice cases brought against orthopedists is necessary.

In China, the medicolegal system is the same in each province, municipality, and autonomous region, and all medical disputes and incidences of malpractice should be handled according to the Medical Malpractice Management Regulation and Regulations on the Prevention and Handling of Medical Disputes. When medical disputes occur in China, the patients and physicians can reach a consensus through the following routes: mutual voluntary negotiation, application to the people’s committee for mediation, application for administrative mediation, and appeals to the people’s court, etc. If the physicians and the patients cannot reach a consensus via mutual voluntary negotiation, the physician’s degree of responsibility regarding the medical malpractice, which can be divided into 5 levels, must be judged before the disputes are handled using the afore-mentioned routes.

The keys to reducing or preventing errors and accidents are acquiring ongoing information on the factors associated with medical malpractice and adopting preventative strategies. Therefore, we performed a cross-sectional, descriptive survey in China on the characteristics relating to medical malpractice cases against orthopedists from 2016 to 2017. We aimed to investigate the medical errors associated with medical malpractice that are made by orthopedists, identify the factors associated with a large degree of responsibility in medical malpractice, and examine the principles that will foster improvement in the quality of patient care and reduce the incidence of orthopedic medical malpractice cases in future.

## Materials and methods

**Fig 1:**
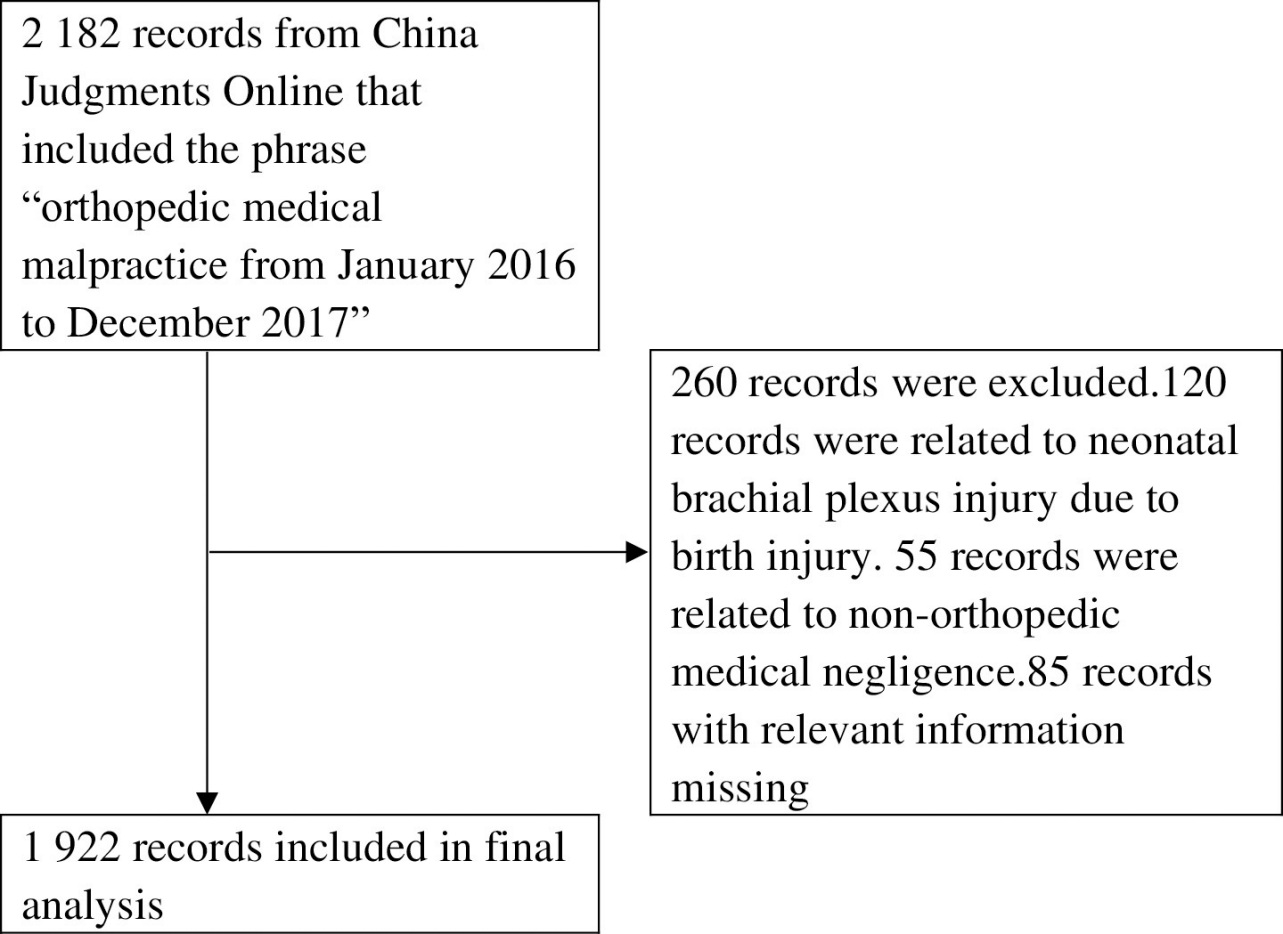
Flowchart of the selection of records of medical litigation in China between 2016 and 2017.

To examine the professional liability risk among orthopedists, we collected data from the Hygiene Administrative Department of the Medical Accident Appraisal Center of the Chinese Medical Association from August 2018. Participants’ identities were not required during or after data collection. Records from which any basic information was missing and non-orthopedic cases were excluded (Fig 1). If 1 case was reported in multiple records, we only kept the record of the final judgement.

The medical malpractice identification process was as follows. First, both doctors and patients submitted appraisal materials, and the Provincial Medical Association composed and stipulated the number of people in the identification team according to the specific medical accident dispute case and each side’s point of view. After performing the necessary avoidance procedures, the Medical Association staff used a computer to randomly select the expert and alternate expert numbers to form the expert identification group. The medical accident technical appraisal meeting was presided over by the expert appraisal group leader. First the patient, then the doctor, stated their opinion and reasons within the prescribed time and answered the expert group’s questions. After an on-the-spot physical examination, the expert group formed the appraisal conclusion according to the consensus of the expert appraisal group members, and the team leader issued the medical accident technical appraisal certificate.

This study was approved by the Institutional Review Board of the Third Hospital of Hebei Medical University in compliance with the Declaration of Helsinki. The requirement for informed consent was waived due to the retrospective nature of the study. This study was authorized by the Chinese Medical Association.

## Indicators/outcome measures

### Patient demographic information

Participants were classified by age into 4 major groups: children (≤14 years), young adults (15–44 years), middle-aged adults (45–64 years), and the elderly (65 years and over).

### Relevant hospital information

The following information was collected: hospital level (3 levels: provincial, city, and county), region (3 regions: eastern, western, and central), province (where the hospital was located), inpatient or outpatient status, interval between the admission and the time of the incident and the seeking of medical expertise (3 intervals: <1 year, 1–2 years, >2 years).

### Relevant injury information

The following information regarding the injury was collected. The injury mechanisms (for traumatic fractures these mechanisms included: traffic accidents; falls from heights; slips, trips, or falls; crush injuries; sharp and blunt force trauma; and no injury). This classification system is used commonly in the field of traumatology and orthopedics. The pathological type (including fracture/dislocation, arthritis, spinal lesions, femoral/humeral head necrosis, soft tissue injuries, tuberculosis, benign tumors, and others), injury number (representing the number of injured parts), injury type (divided into 3 types: open, closed, and no injuries), injury site (including the spine, femur, tibia and fibula, ulna and radius, humerus, foot, hand, knee joint, scapula and clavicle, rib, pelvis and acetabulum, and head) were also noted.

### Relevant treatment information

We collected information on the following aspects of the patient treatment: hospitalization and operation durations, operative type (divided into 4 types: conservative treatment, minimally invasive internal fixation, open reduction internal fixation, external fixation and surgery without internal fixation), and elective or traumatic cases. We also noted the patients’ preoperative comorbidities (2 categories: none or yes) including diabetes; hypertension; cardiovascular, cerebrovascular, respiratory, liver, and renal disease; lower extremity venous thrombosis, and others.

### Medical errors made by the orthopedic surgeon

1. Failure to supervise or monitor a case meant the doctors did not carefully and comprehensively observe the changes in the disease; thus, they failed to provide timely symptomatic treatment measures.
2. Improper procedure performance meant the surgery did not conform to certain specifications, such as aseptic operation specification violations.
3. Failure to instruct or communicate with the patient meant the medical party did not fulfill the obligation of informed disclosure, or that the information was not made available and there was insufficient communication.
4. An escape diagnosis meant that 1 patient had different diseases simultaneously, and the doctor omitted the diagnosis of a certain disease.
5. Incorrect selection of the surgical method meant the wrong surgical method was used, such as improper selection of the internal fixation steel plate, steel nail, or external fixation support.
6. Failure to recognize a treatment complication meant the doctors did not find the complications in time and did not take timely and effective treatment measures.
7. Improper supervision of the residents or other staff; for example, an unqualified doctor operating on the patient.
8. Postoperative anticoagulation and anti-infection measures were not standardized; for example, anticoagulants were not given promptly for patients with thromboembolism, or antibiotics were not used to prevent infection in patients with infection risk.
9. Non-standard medical records meant the medical department did not adequately document the medical records or did not comply with the relevant provisions of the basic standard for writing medical records.
10. Misdiagnosis referred to an incorrect conclusion on the disease.
11. Medication errors meant incorrect drug use, such as administering anticoagulants to patients at risk of bleeding or stopping anticoagulants prematurely after surgery.
12. Hospitals without the relevant surgical qualifications; for example, when a hip replacement was performed at an unqualified hospital.
13. Procedure was performed when it was not indicated or contraindicated included indications that were not strictly controlled, improper timing of the operation, or not ruling out contraindications.
14. Anesthesia problems included an improper choice of anesthetic method, or nonstandard use of narcotic drugs and anesthetic management.

### The physician’s degree of responsibility regarding the medical negligence

The physician’s degree of responsibility regarding the medical negligence was divided into 5 levels: 1. The physician was fully responsible, which meant that the damage to the patient from the medical malpractice was completely caused by the physician or surgeon, who bore full responsibility. 2. Primary responsibility of the physician meant that the damage to the patient due to the medical malpractice was mainly caused by the physician or surgeon, and other factors played a secondary role. 3. The physician bore equal responsibility referred to the combined action of medical negligence and other factors, and the physician or surgeon was approximately 50% responsible for the damages that the patient suffered. 4. Secondary responsibility of the physicians meant that the physician or surgeon was partially responsible for the damages that the patient suffered and medical negligence played a secondary role in the impairment. 5. Minor responsibility of the physician meant that the majority of the consequences related to the medical negligence were caused by other factors than the physician. The patients were divided into 2 groups: Group A, including minor and secondary responsibility, and Group B, including equal, primary, and full responsibility. Logistic regression analysis was used to compare the relevant information such as the patient demographics, hospital level, injury, treatment, and orthopedists’ skill level between the 2 groups.

### Statistical analysis

The *χ*^*2*^ test was used to compare the differences between Groups A and B in the nominal variable categories, such as sex; inpatient/outpatient status; preoperative comorbidities; interval between hospital stay and request for medical expertise; hospital level, region, and province; injury cause; pathological type; injury site and type; operation type; and medical error. Multiple classification logistic regression models were constructed to explore how the potential factors for medical malpractice related to the degree of responsibility. Variables for which the *P*-value was ≤0.20 in the univariate analysis were included as candidate variables in the multivariate models. All statistical analyses were performed using SPSS version 25.0 (IBM Corp., Armonk, NY, United States).

## Results

### Age- and sex-specific characteristics

During the study period, a total of 1922 medical malpractice cases met the inclusion criteria, of which 1195 and 727 (62.2% and 37.8%, respectively) were men and women, respectively. The average patient age was 48.5±15.2 years old. Every patient had poor outcomes after treatment, such as amputation, joint dysfunction, paralysis, and death. The proportions of the different age groups from high to low were 62.5%, 21.1%, 12.5%, and 3.9% (1202, 406, 240, and 75 cases, respectively) for middle-aged adults, young adults, the elderly, and children, respectively (Table 1).

**Table 1:** Case characteristics of orthopedist’s degree of responsibility for medical malpractice in China between 2016 and 2017.

Issues	*n*	(%)	Full/Major/Equal/Secondary/Minor	Equal and above/ secondary and minor	*χ*^*2*^ value	*P* value
Age						
≤14	74	(3.9)	10/31/0/23/10	41/33	27.530	0.006*
15~44	406	(21.1)	33/141/12/165/55	186/220		
45~64	1202	(62.5)	81/407/57/424/233	545/657		
≥65	240	(12.5)	11/75/8/104/42	94/146		
Gender						
Male	1195	(62.2)	76/392/46/470/211	514/681	5.335	0.021*
Female	727	(37.8)	59/262/31/246/129	352/375		
Hospital level						
Province-level	199	(10.4)	11/54/7/83/44	72/127	12.899	0.002*
City-level	1006	(52.3)	62/131/56/385/182	439/567		
County-level	717	(37.3)	62/279/14/248/114	355/362		
Region						
Eastern	1001	(52.1)	71/299/64/366/201	437/567	5.839	0.050
Central	397	(20.7)	36/155/9/142/55	200/197		
Western	524	(27.3)	28/200/4/208/84	232/292		
Inpatient/outpatient						
Inpatient	1810	(94.2)	122/615/69/681/323	806/1004	3.483	0.060
Outpatient	112	(5.8)	13/39/8/35/17	60/52		
Interval between the first onset and medical expertise						
<1 year	783	(40.7)	51/275/32/290/135	358/425	2.275	0.320
1–2 years	646	(33.6)	52/219/29/241/105	300/346		
>2 years	493	(25.7)	32/160/16/185/100	208/285		
Injury causes						
Traffic accident	365	(19.0)	12/103/20/156/74	135/230	22.998	0.001*
Slip, Trip or Fall	654	(34.2)	52/220/22/244/116	294/360		
Fall from Heights	54	(2.8)	0/14/3/30/7	17/37		
Crushing injury	130	(6.8)	4/48/4/51/23	56/74		
Sharp trauma	82	(4.3)	9/30/2/27/14	41/41		
Blunt force trauma	72	(3.7)	5/29/1/23/14	35/37		
No injury	565	(29.2)	53/210/25/185/92	288/277		
Pathological types						
Fracture/dislocation	1229	(63.9)	74/404/52/479/220	530/699	18.790	0.009*
Arthritis	89	(4.6)	8/24/4/34/19	36/53		
Spinal degenerative disease	253	(13.2)	20/97/10/84/42	127/126		
Femoral/humeral head necrosis	36	(1.9)	4/17/2/9/4	23/13		
Soft tissue injuries	71	(3.7)	8/31/3/16/13	42/29		
Skeletal tuberculosis	158	(8.2)	12/49/4/63/30	65/93		
Benign tumors	52	(2.7)	6/21/1/18/6	28/24		
Others(developmental malformations, gout stones, deletions, tumors)	34	(1.8)	3/11/1/13/6	15/19		
Injury numbers						
1.00	1637	(85.2)	125/566/63/602/281	754/883	7.393	0.025*
2.00	205	(10.7)	7/73/7/76/42	87/118		
≥3	80	(4.2)	3/15/7/38/17	25/55		
Injury types						
Open injury	304	(15.8)	14/83/9/141/57	106/198	20.671	<0.01*
No injury	580	(30.2)	67/355/43/383/190	465/573		
Closed injury	1038	(54.0)	54/216/25/192/93	295/285		
Times of hospitalization						
Once	50	(2.6)	6/14/1/16/13	21/29	0.210	0.980
Twice	775	(40.3)	70/251/29/280/145	350/425		
Three times	667	(37.4)	39/240/23/253/112	302/365		
≥4 times	430	(22.4)	20/149/24/167/70	193/237		
Times of operation						
Once	201	(10.5)	18/54/11/79/39	83/118	1.592	0.450
Twice	1328	(69.1)	101/462/46/480/239	609/719		
>twice	393	(20.4)	16/138/20/157/62	174/219		
Operative type						
Conservative treatment	364	(18.9)	49/290/33/317/158	372/475	16.043	0.003*
Minimally invasive internal fixation	210	(10.9)	49/168/20/150/68	237/218		
Open reduction internal fixation	847	(44.1)	24/105/17/144/74	146/218		
External fixation	46	(2.4)	11/79/6/87/27	96/114		
Surgery without internal fixation	455	(23.7)	2/12/1/18/13	15/31		
Elective/trauma					5.558	0.018*
Traumatic case	1441	(75.0)	94/475/58/553/261	627/814		
Elective case	481	(25.0)	41/179/19/163/79	239/242		
Preoperative comorbidities						
Yes	414	(21.5)	22/117/24/175/76	163/251	6.889	0.009*
No	1508	(78.5)	113/537/53/541/264	703/805		

*Significant at α = 0.05.

### Relevant hospital characteristics

Among the 1922 medical malpractice cases, there were 1006 that occurred at a city level hospital that accounted for 52.3% of the cases, followed by county and provincial level cases (717 and 199 cases, 37.3% and 10.4%, respectively). The majority of cases involved inpatients with a 1-year interval between symptom onset and the request for medical expertise (1810 and 783 cases; 94.2% and 40.7%, respectively). There were 1001 cases that occurred in the eastern region, accounting for 52.1% of all cases, followed by the western and central regions (524 and 397 cases, 27.3% and 20.7%, respectively; Table 1). Orthopedic medical malpractice often occurred in the Jiangsu and Zhejiang provinces (279 and 233 cases, 14.52% and 12.12%, respectively; Table 2).

**Table 2:** Provinces of orthopedic medical malpractice in China between 2016 and 2017.

Provinces	*n*	(%)
**Anhui, central China**	93	(4.84)
**Beijing, eastern China**	7	(0.36)
**Chongqing, western China**	1	(0.05)
**Fujian, eastern China**	42	(2.19)
**Gansu, western China**	8	(0.42)
**Guangdong, eastern China**	13	(0.68)
**Guizhou, western China**	168	(8.74)
**Hebei, eastern China**	84	(4.37)
**Heilongjiang, central China**	44	(2.29)
**Henan, central China**	52	(2.71)
**Hubei, central China**	81	(4.21)
**Hunan, central China**	52	(2.71)
**Jiangsu, eastern China**	279	(14.52)
**Jiangxi, central China**	30	(1.56)
**Jilin, central China**	33	(1.72)
**Liaoning, eastern China**	100	(5.20)
**Qinghai, western China**	1	(0.05)
**Shanxi, western China**	29	(1.51)
**Shandong, eastern China**	47	(2.45)
**Shanghai, eastern China**	139	(7.23)
**Shanxi, central China**	12	(0.62)
**Sichuan, western China**	97	(5.05)
**Tianjin, eastern China**	57	(2.97)
**Yunnan, western China**	87	(4.53)
**Zhejiang, eastern China**	233	(12.12)
**Autonomous regions**		
**Guangxi, western China**	5	(0.26)
**Inner Mongolia, western China**	64	(3.33)
**Ningxia, western China**	1	(0.05)
**Xinjiang, western China**	63	(3.28)

### Relevant injury characteristics

Among the 1922 medical malpractice cases, there were 654 cases (34.2%) of low energy injuries, followed by no injuries and traffic accidents (565 and 365 cases, 29.2% and 19.0%, respectively). There were 1229 cases of fractures/dislocations, accounting for 63.9%, followed by spinal degenerative disease and skeletal tuberculosis (253 and 158 cases, 13.2% and 8.2%, respectively). Most patients had 1 injury (1637 cases, 85.2%). Among the injury types, closed injuries (1038 cases, 54.0%) were the most common (Table 1). Among the injury sites, spine and femur injuries were the most common (398 and 366 cases, 20.7% and 19.0%, respectively; Table 3).

**Table 3:** Orthopedist’s degree of responsibility for medical malpractice according to injury sites in China between 2016 and 2017.

Issues	*n*	(%)	Full/Major/Equal/Secondary/Minor	Equal and above/ Secondary and Minor	*χ*^*2*^ value	*P* value
Spine						
Yes	398	(20.7)	25/151/17/140/65	193/205	2.393	0.122
No	1524	(79.3)	110/503/60/576/275	673/851		
Femur						
Yes	366	(19.0)	20/116/14/164/52	150/216	3.030	0.082
No	1556	(81.0)	115/538/63/552/288	716/840		
Tibia and fibula						
Yes	290	(15.1)	8/79/12/134/57	99/191	16.449	<001*
No	1632	(84.9)	127/575/65/582/283	767/865		
Ulna and radius						
Yes	233	(12.1)	10/91/5/80/47	106/127	0.020	0.886
No	1689	(87.9)	125/563/72/636/293	760/929		
Humerus						
Yes	195	(10.1)	23/72/14/54/32	109/86	10.301	0.001*
No	1727	(89.9)	112/582/63/662/308	757/970		
Foot						
Yes	185	(9.6)	13/65/9/66/32	87/98	0.321	0.571
No	1737	(90.4)	122/589/68/650/308	779/958		
Hand						
Yes	158	(8.2)	12/59/2/58/27	73/85	0.091	0.763
No	1764	(91.8)	123/595/75/658/313	793/971		
Knee joint						
Yes	145	(7.5)	10/47/5/51/32	62/83	0.335	0.563
No	1777	(92.5)	125/607/72/665/308	804/973		
Scapular and clavicle						
Yes	104	(5.4)	9/30/8/37/20	47/57	0.001	0.977
No	1818	(94.6)	126/624/69/679/320	819/999		
Rib						
Yes	79	(4.1)	5/17/7/35/15	29/50	2.319	0.128
No	1843	(95.9)	130/637/70/681/325	837/1006		
Pelvis and acetabulum						
Yes	74	(3.9)	10/21/4/25/14	35/39	0.156	0.693
No	1848	(96.1)	125/633/73/691/326	831/1017		
Head						
Yes	27	(1.4)	2/3/1/15/6	6/21	5.768	0.016*
No	1895	(98.6)	133/651/76/701/334	860/1035		

*Significant at α = 0.05.

### Relevant treatment characteristics

Most of the patients were treated with surgery (1558 cases, 81.1%), most of which were open reduction and internal fixation (847 cases, 44.1%). Most were trauma cases, hospitalized twice, and underwent 2 surgeries (1441, 775, and 1328 cases; 75.0%, 40.3%, and 69.1%, respectively). There were 414 patients (21.5%) who had preoperative comorbidities (Table 1).

### Medical errors made by the orthopedic surgeon

Most medical errors that were made by orthopedists in the medical malpractice cases involved failure to supervise or monitor the case (738 cases, 38.4%). There were fewer cases of medical error that involved improper performance of procedures and failure to instruct or communicate with the patient (721 and 425 cases, 22.1% and 37.5%, respectively; Table 4).

**Table 4:** Medical error of orthopedist’s degree of responsibility for medical malpractice in China between 2016 and 2017.

Issues	*n*	(%)	Full/Major/Equal/Secondary/Minor	Equal and above/ Secondary and Minor	*χ*^*2*^ value	*P* value
Failure to supervise or monitor case						
Yes	736	(38.3)	51/262/38/254/131	351/385	3.340	0.068
No	1186	(61.7)	85/391/39/459/212	515/671		
Improper performance of procedures						
Yes	721	(37.5)	48/252/26/269/126	326/295	0.012	0.914
No	1201	(62.5)	87/402/51/447/214	540/661		
Failure to instruct or communicate with patient						
Yes	425	(22.1)	22/148/18/157/80	188/237	0.149	0.700
No	1497	(77.9)	113/506/59/559/260	678/819		
Escape diagnosis						
Yes	255	(13.3)	20/81/10/91/53	111/144	0.277	0.599
No	1667	(86.7)	115/573/67/625/287	755/912		
Incorrect selection of operation method						
Yes	244	(12.7)	18/80/7/97/42	105/139	0.463	0.496
No	1678	(87.3)	117/574/70/619/298	761/917		
Failure to recognize a complication of treatment						
Yes	154	(8.0)	11/61/6/58/18	78/76	2.115	0.146
No	1768	(92.0)	124/593/71/658/322	788/980		
Improper supervision of residents or other staff						
Yes	130	(6.8)	5/45/3/56/21	53/77	1.036	0.309
No	1792	(93.2)	130/609/74/660/319	813/979		
Yes	90	(4.7)	5/31/4/34/16	40/50	0.014	0.905
No	1832	(95.3)	130/623/73/682/324	826/1006		
Non-standard medical records						
Yes	89	(4.6)	4/32/4/31/18	40/49	0.001	0.982
No	1833	(95.4)	131/622/73/685/322	826/1007		
Misdiagnosis						
Yes	77	(4.0)	10/25/3/28/11	38/39	0.597	0.440
No	1845	(96.0)	125/629/74/688/329	828/1017		
Medication errors						
Yes	46	(2.4)	3/13/1/20/9	17/29	1.249	0.264
No	1876	(97.6)	132/641/76/696/331	849/1027		
Hospitals without relevant surgical qualifications						
Yes	27	(1.4)	3/10/0/11/3	13/14	0.106	0.745
No	1895	(98.6)	131/646/76/709/333	853/1042		
Procedure performed when not indicated or contraindicated						
Yes	27	(1.4)	4/8/1/7/7	13/14	0.106	0.745
No	1895	(98.6)	131/646/76/709/333	853/1042		
Anesthesia problem						
Yes	16	(0.8)	0/9/0/6/1	9/7	0.816	0.366
No	1906	(99.2)	135/645/77/710/339	857/1049		

*Significant at α = 0.05.

### The physician or surgeon’s degree of responsibility regarding the medical negligence

Between 2016 and 2017, the most frequent degree of responsibility in the medical malpractice claims against orthopedists was secondary responsibility (716 cases, 37.3%). There were fewer cases that involved the following degrees of responsibility: full, primary, equal and minor responsibility (135, 654, 77 and 340 cases; 7.0%, 34.0%, 4.0%, and 17.7%, respectively; Table 2). There were 1315, 314, 151, 90, 34, 14, 3, and 1 orthopedists (68.4%,16.3%, 7.9%, 4.7%, 1.8%, 0.7%, 0.2%, and 0.1%, respectively) who made 1, 2, 3, 4, 5, 6, 7, and 8 medical errors in the diagnosis and treatment, respectively.

### Factors associated with the degree of responsibility in orthopedic medical malpractice cases

There were 866 orthopedic surgeons who had an equal or higher degree of responsibility in the medical malpractice case, and 1056 who had a secondary or lower degree of responsibility. Univariate analysis showed that there were significant differences in the degrees of liability that the orthopedist bore in the medical malpractice case depending on the patient characteristics, such as the sex; age; injury type; preoperative comorbidities; cause of injury; operation type; hospital level; pathological type; number of injuries; fracture of the tibia and fibula, humerus, and head; and the medical errors of failure to supervise or monitor a case and recognize a treatment complication (*P*<0.05).

Table 5 summarizes the factors associated with an equal and higher degree of responsibility in medical malpractice cases. Compared with patients aged 0–14 years, those aged ≥65 years were more likely to claim that the orthopedic doctor bore a serious degree of responsibility for the medical malpractice (odds ratio [*OR*] = 1.801; 95% confidence interval [CI], 1.019–3.182; *P* = 0.043). Patients with preoperative comorbidities were more likely to claim that the orthopedic doctor bore a serious degree of responsibility for the medical malpractice than those who did not have preoperative comorbidities (*OR* = 1.340; 95% CI, 1.045–1.718; *P* = 0.021). Compared with conservative treatment, undergoing surgical treatment without internal fixation had a protective effect against the orthopedic doctor bearing a serious degree of responsibility for the medical malpractice (*OR* = 0.632; 95% CI, 0.460–0.869; *P* = 0.005). Using county level hospitals as a reference, being treated at the provincial or city level increased the likelihood of the orthopedic doctor bearing a serious degree of responsibility for the medical malpractice (*OR* = 1.790 and 1.296; 95% CI, 1.270–2.524 and 1.052–1.596; *P* = 0.001 and *P* = 0.015, respectively). Patients with humerus injuries and those treated by doctors who failed to supervise or monitor them (*OR* = 1.478 and 1.223; 95% CI, 1.058–2.064 and 1.008–1.485; *P* = 0.022 and *P* = 0.041, respectively) were prone to claim that the orthopedic doctor bore a serious degree of responsibility for the medical malpractice (Table 5).

**Table 5:** Multivariate analysis of the factors associated with orthopedist’s degree of responsibility for medical malpractice in China between 2016 and 2017.

Predictor	Estimate	Std. Error	Wald	*P* value	*OR* value	95%CI		
**Age (years)**			5.559	0.135				
**0–14**	Reference							
**15–44**	0.230	0.270	0.727	0.394	1.258	0.742	~	2.134
**45–64**	0.304	0.257	1.394	0.238	1.355	0.818	~	2.244
**≥65**	0.588	0.290	4.104	0.043*	1.801	1.019	~	3.182
**Preoperative complications**								
**No**	Reference							
**Yes**	0.292	0.127	5.314	0.021*	1.340	1.045	~	1.718
**Operative type**			8.861	0.065				
**Conservative treatment**	Reference							
**Open reduction internal fixation**	-2.393	0.188	1.624	0.202	0.787	0.545	~	1.137
**Minimally invasive internal fixation**	-0.255	0.141	3.251	0.071	0.775	0.588	~	1.022
**External fixation**	0.032	0.349	0.009	0.927	1.033	0.521	~	2.046
**Surgery without internal fixation**	-0.458	0.162	7.997	0.005*	0.632	0.460	~	0.869
**Hospital level**			12.776	0.002				
**County-level**	Reference							
**Province-level**	0.582	0.175	11.036	0.001*	1.790	1.270	~	2.524
**City-level**	0.259	0.106	5.933	0.015*	1.296	1.052	~	1.596
**Humerus injury**								
**No**	Reference							
**Yes**	0.391	0.171	5.246	0.022*	1.478	1.058	~	2.064
**Failure to supervise or monitor case**								
**No**	Reference							
**Yes**	0.204	0.099	4.268	0.041*	1.223	1.008	~	1.485

*Significant at α = 0.05.

## Discussion

To the best of our knowledge, this study is the first clinically verified, comprehensive, national investigation of medical malpractice cases against orthopedists performed in China. The orthopedics department is a unique environment that performs a large volume of operations and has multiple complex factors that contribute to potential errors. It is important for orthopedic surgeons to understand these factors associated with medical malpractice. This study identified the most frequent causes of medical malpractice against orthopedists and determined the plaintiff demographics, clinical characteristics, and factors associated with medical malpractice.

Increasing numbers of medical malpractice claims have been observed in both Western countries [5] and China [6]. In this study, there were 1922 orthopedic medical malpractice cases in China from 2016 to 2017. All the cases were claims in which the final judgments were against the physician and were consistent with the final court judgment. In this study, the most common anatomical sites involved in orthopedic medical malpractice cases were the spine and femur, there were more men than women, more inpatients than outpatients, and more city level hospitals involved than county and provincial level hospitals. However, the results differed when compared with other countries. A general ageing of the Italian population has been recorded in recent years, the most commonly involved anatomical sites were the hips and knees [7]. A comprehensive, nationwide analysis of medical malpractice litigation following total joint arthroplasty found that cases were more common in women than men [8]. Our results were similar to those of many other studies. A retrospective review of fracture-related malpractice lawsuits from 1988 to 2015 in America showed that the mean age of the plaintiff was 48.5±15.2 years and the most commonly involved anatomical sites were the spine and femur [9]. Klimo et al. [10] reported that the lumbar spine was the most common anatomic area involved in orthopedic medical malpractice cases. Zhang et al. [11] found that medical malpractice mainly occurred at tertiary medical institutions, followed by secondary medical institutions. Lu [12] reported that more men were involved in medical malpractice cases.

In our study, most medical malpractice cases occurred in the eastern part of China (52.1%), including Jiangsu and Zhejiang (14.52% and 52.1%, respectively). Medical malpractice is very susceptible to the policy. The medical malpractice system in Western countries differs from that in China. The medical malpractice systems in the United States and United Kingdom are based on British common law. Each state in the United States has its own medical malpractice laws that differ between the states. The malpractice rates can vary greatly depending on the differing interstate laws. Contrastingly, in China, the medicolegal system is the same in each province, municipality, and autonomous region, and all medical disputes and malpractice claims are handled according to the Interim Measures for Medical Accident Technical Appraisal [13], Regulations on the Prevention [14] and Handling of Medical Disputes and Medical Malpractice Management Regulations [15]. Fault identification for medical accidents can be performed by medical associations or judicial authentication institutions according the above-mentioned laws, however, different provinces may differ in selecting medical associations or judicial authentication institutions. Before July 2017, the fault identification in medical malpractice cases in Jiangsu province were performed by local medical associations without judicial appraisal. On December 22, 2017, the Jiangsu Higher People’s Court, Jiangsu Health Commission, and Department of Justice of Jiangsu Province mutually issued The Administrative Management Regulations of Medical Damage Appraisal in Jiangsu Province in which the use of judicial or forensic medical appraisal was first allowed [16]. This is similar to Zhejiang province, where all the faults in medical malpractice cases are also identified by local medical associations with no judicial appraisal; however, other provinces have 2 ways to identify who takes responsibility in medical malpractice claims: local medical associations or judicial authentication institutions. This may be why we found more cases in Jiangsu and Zhejiang provinces; however, these findings may not have been a true reflection of the situation.

This study found that the number of medical malpractice cases were higher in inpatients and traumatic cases than those for outpatients and elective cases because they are more complex, urgent, or difficult to treat. This study also found that most medical malpractice cases occurred in patients with closed injuries and those who underwent open reduction and internal fixation. This is because closed injuries are easy to misdiagnose, and patients who undergo open reduction and internal fixation are more likely to have greater trauma, more comorbidities, and longer hospitalizations. In this study, the most common degree of responsibility in medical malpractice claims against orthopedists was secondary, which was consistent with the findings from other studies [17].

This comprehensive study of medical malpractice in the field of orthopedics between 2016 and 2017 found that most orthopedists’ medical errors involved failure to supervise or monitor a case, improper performance of procedures, and failure to instruct or communicate with the patient. These findings are similar to those from many previous studies [18, 19]. This is the easiest medical malpractice prevention measure to develop. Improved communication, patient monitoring and orthopedist education can significantly reduce the number of medical malpractice incidents [20]. It is essential that the physician-in-charge should personally manage and communicate with patients. Honest communication that the patient can understand and frequent monitoring will help keep the patient from feeling abandoned. Training programs, such as those that explain operation standards, could be used to improve how these surgical operation errors are avoided. More formal tracking of written or verbal information provided to patients can prevent unannounced patients. When a serious diagnosis or procedure is explained, the patient may struggle to pay attention [21–23]. Some simple measures can be implemented in clinical work. Measures such as developing a treatment passport or information sessions with doctors and other healthcare staff can improve patients’ grasp of the information. Written materials or multimedia presentations can improve patients’ understanding and compliance [24, 25].

Another issue elucidated in this study was that we explored the potential factors associated with a serious degree of responsibility for orthopedic doctors in medical malpractice cases. When compared with participants aged 0–14 years, those aged ≥65 years were more likely to claim that the physician bore a serious degree of responsibility. These findings are similar to those of some previous studies that showed that older patients had multiple preoperative comorbidities and poor outcomes after orthopedic surgery [26–28].

Preoperative comorbidities were associated with claims that the orthopedic doctor bore a serious degree of responsibility, which may be due to their association with more complex conditions and treatments. Our data suggested that, when compared with conservative treatment, undergoing surgical treatment without internal fixation had a protective effect against claims that the orthopedic doctor bore a serious degree of responsibility for the medical malpractice. This was consistent with what we observed clinically. Patients who underwent conservative fracture treatment were prone to displacement at later stages, which increased the medical malpractice risk. When county level hospitals were used as a reference, another issue that this study noted was that being treated at a provincial or city level hospital increased the likelihood of claims that the orthopedic doctor bore a serious degree of responsibility for the medical malpractice. This may be because provincial and city level hospitals are both more likely to receive and manage seriously injured patients. Other factors associated with a serious degree of responsibility in medical malpractice were humerus injuries and failure to supervise or monitor a case. These findings were consistent with those of other studies [18, 19, 29, 30]. For example, Festge et al. [29] conducted an analysis of the complaints concerning 173 cases of fractures and dislocations of the upper extremity in children. They concluded that humerus fractures were one of the major causes of medical malpractice. Singh et al. [30] conducted a study of closed malpractice claims from 5 insurers, elucidating an important problem regarding a lack of supervision that was prevalent with types of teamwork. In addition, lack of supervision was the most prevalent type of problem. Graduate medical education reform should focus on strengthening these aspects of training.

Although this study is the first clinically verified, comprehensive, nationwide investigation of medical malpractice cases against orthopedists performed in China, there are some limitations that should be considered. First, it was retrospective with a limited number of cases. This may have led to less accurate information. We reduced this bias by conducting our study on data that was checked repeatedly by the Hygiene Administrative department of the Medical Accident Appraisal Center of the Chinese Medical Association. Second, detailed information regarding the healthcare providers (surgeons and/or nurses) was not available. Third, the cases that underwent judicial authentication and that involved private negotiating on a voluntary basis between healthcare providers and patients without any official medical fault identification procedures were not included in this study. Fourth, without a denominator many data were difficult to interpret.

## Conclusions

Our results provide detailed information on the plaintiff demographics, clinical characteristics, and the factors associated with medical malpractice that can now be used as an up-to-date clinical evidence base for national healthcare planning and preventive efforts in China and elsewhere. Medical malpractice is related to the effects of poor treatment; however, it is not a sufficient condition for medical malpractice. The first measure to prevent medical malpractice is to comprehensively improve the quality of medical personnel, mainly through medical ethics cultivation, professional ability training, and improvement of treatment techniques, which are the key to the prevention and control of the occurrence of errors and accidents. Failure to supervise or monitor a case was the most common cause of medical malpractice, as well as one factor for orthopedists to bear a higher degree of responsibility for the medical malpractice. Orthopedists should enhance the amount of supervision, especially in the treatment of older patients, those with more preoperative comorbidities, and those with humerus injuries.

## Supporting information

S1 Data (XLSX)
